# Retraction notice for: “Comparison of Arndt-endobronchial blocker plus laryngeal mask airway with left-sided double-lumen endobronchial tube in one-lung ventilation in thoracic surgery in the morbidly obese” [Braz J Med Biol Res (2018) 51(2): e6825]

**DOI:** 10.1590/1414-431X20176825retraction

**Published:** 2018-08-16

Z.J. Zhang, M.L. Zheng, Y. Nie and Z.Q. Niu

Department of Anesthesiology, the Cangzhou Central Hospital, Cangzhou, Hebei, China

**Retraction for:** Braz J Med Biol Res | doi: 10.1590/1414-431X20176825 | PMID: 29267506

The Brazilian Journal of Medical and Biological Research was contacted by one specialist questioning the validity of this study. [Click here to view Specialist message]

The authors were contacted on May 7 and 22, 2018 to answer the questions one-by-one. As of June 29, 2018, we did not receive an answer from the authors.

The Editors decided to Retract the article: “Comparison of Arndt-endobronchial blocker plus laryngeal mask airway with left-sided double-lumen endobronchial tube in one-lung ventilation in thoracic surgery in the morbidly obese” that was published in volume 51 no. 2 (2018) (Epub Dec 18, 2017) in the Brazilian Journal of Medical and Biological Research <10.1590/1414-431X20176825> PMID: 29267506.

The Brazilian Journal of Medical and Biological Research informed all authors before the publication of this Retraction.

